# Radiomics model based on coronary CT angiography for predicting major adverse cardiovascular events in patients with coronary artery disease: comparison of lesion-specific pericoronary adipose tissue model and pericoronary adipose tissue model

**DOI:** 10.3389/fcvm.2025.1600942

**Published:** 2025-10-14

**Authors:** Ziguang Huang, Jianing Chen, Huan Ding, Haoyan Pan, Zhaoyuan Xing, Lijuan Zhao, Jing Wen, Zhe Zhang, Baoying Zhao, Xu Dai

**Affiliations:** ^1^ The First Clinical College, Liaoning University of Traditional Chinese Medicine, Shenyang, Liaoning, China; ^2^ CT Clinical Science, Philips Healthcare, Shenyang, Liaoning, China; ^3^ Department of Radiology, The Affiliated Hospital of Liaoning University of Traditional Chinese Medicine, Shenyang, Liaoning, China

**Keywords:** coronary computed tomography angiography, pericoronary adipose tissue, lesion-specific pericoronary adipose tissue, radiomics, major adverse cardiovascular events

## Abstract

**Objective:**

To assess the performance of a lesion-specific pericoronary adipose tissue (PCAT) radiomics model in comparison to a right coronary artery (RCA) PCAT model in predicting major adverse cardiovascular events (MACE) over a three-year period in patients diagnosed with coronary artery disease (CAD). Additionally, the study aims to evaluate the incremental predictive value of combined models integrating clinical features.

**Methods:**

This study conducted a retrospective analysis involving 242 patients with coronary artery disease who underwent coronary CT angiography (CCTA) with MACE occurring in 121 cases. The right coronary artery and lesion-specific PCAT were segmented using the Peri-coronary Adipose Tissue Analysis Tool software (Shukun Technology Co., Ltd.), and 93 radiographic features were extracted, and the features were screened by Pearson correlation coefficients and Lasso regression after the features were processed by Min-Max Normalization. Machine learning techniques were utilized to construct four models: the right coronary artery PCAT model (RCA-model), the lesion-specific PCAT model (LS-model), the clinical model (Cli-model), and two combined models (Cli-RCA model and Cli-LS model). The performance of these models was evaluated by receiver operating characteristic (ROC) curves, calibration curve and decision curve analysis (DCA).

**Results:**

The LS-model demonstrated superior predictive performance with AUC values of 0.821 and 0.838 in the training and test cohorts, respectively. This performance surpassed that ofthe RCA-model, which recorded AUC values of 0.789 and 0.788. Notably, the Cli-LS model achieved the highest AUCs of 0.873 and 0.877. The difference in AUC was statistically significant (*p* < 0.05). Calibration curves indicated excellent agreement between predicted and observed risks, as indicated by aHosmer-Lemeshow test result of *P* > 0.05. Furthermore, decision curve analysis confirmed a higher net clinical benefit.

**Conclusion:**

Lesion-specific PCAT radiomics features demonstrate superior predictive capability for MACE compared to f RCA-based features. Integrating clinical risk factors further enhances model performance, offering a noninvasive imaging tool for risk stratification in patients with CAD.

## 1 Introduction

Coronary artery disease (CAD) is recognized as one of the leading lethal factors worldwide (1), leading to endpoint events, such as acute myocardial infarction and sudden death. These outcomes not only constitute a major threat to the lives of patients, but also significantly affect their quality of life. In 1999, Professor Rose had proposed that atherosclerosis is a chronic inflammatory lipid disease (2), with the lipid infiltration theory serving as the pathophysiological foundation of atherosclerosis (3). Furthermore, vascular inflammation plays an important role in the formation, progression, and rupture of atherosclerotic plaques (4). There is a bidirectional communication mechanism between pericoronary adipose tissue (PCAT) and the vessel wall (5). Under pathological conditions, when vascular inflammation occurs, PCAT induces mesenchymal changes in the vessel wall from the outside in through the release of pro-inflammatory factors and inflammatory cytokines. Conversely, the vessel wall can influence PCAT through paracrine secretion. This interplay contributes to the formation of coronary atherosclerotic plaques and facilitates a reversible shift of PCAT from a lipid phase to an aqueous phase, which can be noninvasively identified by imaging techniques such as coronary computed tomography angiography (CCTA).

CCTA has been an important tool for the noninvasive diagnosis of CAD, and plays a crucial role in the assessment of MACE (6). The CRISP CT study (7) demonstrated that the fat attenuation index (FAI) in the periphery of the right coronary artery (RCA) could be used as a representative biomarker for the comprehensive assessment of coronary inflammation and high FAI values are an important indicator of increased cardiac mortality. A study (8) suggested that lesion-specific pericoronary adipose tissue CT attenuation (PCATa) in the region of coronary lesions was better than that in the right coronary artery in predicting MACE. That is, in prognostic evaluation, lesion-specific inflammation has a higher priority compared to overall inflammation.

With the continuous advancement of artificial intelligence technology, the application of radiomics in the field of medical research is expanding. In addition to coronary inflammation due to PCAT, radiomics can also capture spatial structural changes of irreversible PCAT, such as lipofibrosis and microvascular remodeling (9). Numerous studies have confirmed the superiority of radiomics of PCAT in the prediction of adverse cardiovascular events (10–12), and the radiomics model of PCAT in the RCA is superior to the radiomics model of PCAT in the other two coronary arteries (13). It has also been demonstrated (14) that imaging histology models of lesion-specific PCAT have a higher value than traditional risk factors in MACE prediction.

Therefore, we constructed five predictive models: right coronary artery PCAT radiomics model(RCA-model), lesion-specific PCAT radiomics model(LS-model), clinical model(Cli-model), clinical-right-coronary-artery PCAT radiomics model(Cli-RCA model), and clinical-lesion-specific PCAT radiomics model(Cli-LS model). The objective of these models is to investigate the predictive effects of right coronary artery PCAT radiomics characteristics, lesion-specific PCAT radiomics characteristics, and clinical characteristics on the occurrence of MACE within three years following CCTA examination,and to see whether lesion-specific PCAT radiomic feature have incremental value for MCAE prediction.

## 2 Materials and methods

### 2.1 Study population

**Figure 1:**
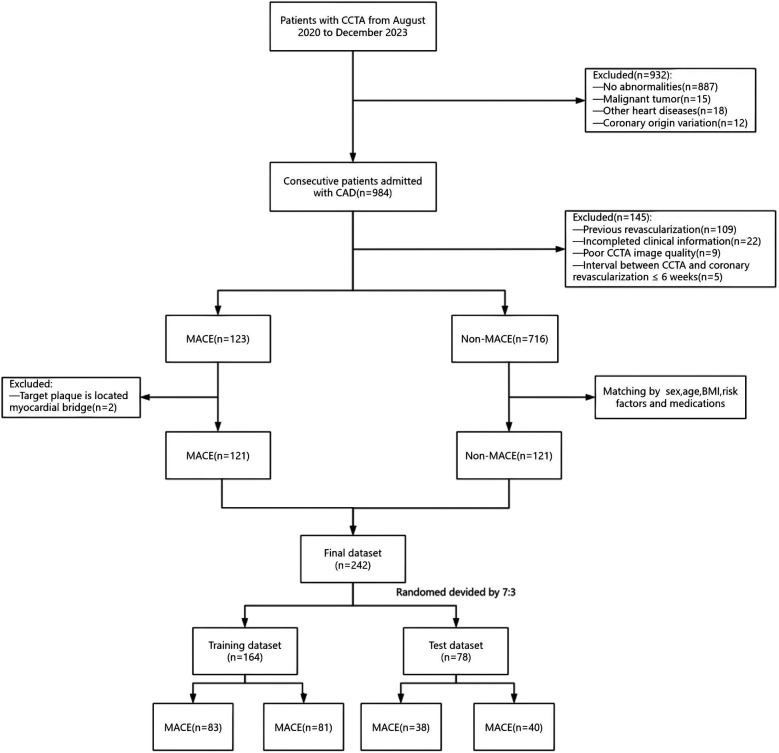
A flowchart of patient recruitment and study design. CCTA, coronary computed tomography angiography; CAD, coronary artery disease; MACE, major adverse cardiovascular events.

This study retrospectively collected 984 patients diagnosed with CAD who underwent CCTA at the Medical Imaging Center of Liaoning University of Traditional Chinese Medicine Hospital from August 2020 to December 2023. Among these patients, 123 patients experienced MACE within three years after undergoing inclusion and exclusion criteria screening. The occurrence of MACE in patients was determined based on the hospital's electronic medical records as well as telephone follow-ups. Clinical risk factors available for each patient were collected, including gender, age, body mass index, cardiovascular risk factors, medication usage, total cholesterol, triglycerides, high-density lipoprotein cholesterol, low-density lipoprotein cholesterol, C-reactive protein, and blood glucose. MACE was defined as cardiac death (including fatal acute myocardial infarction), malignant arrhythmias, nonfatal acute myocardial infarction, new-onset congestive heart failure, coronary revascularization (after six weeks of CCTA), and readmission due to unstable angina. Patients without MACE were matched to patients with MACE in terms of sex, age, body mass index, cardiovascular risk factors, and medications. In this study, multivariate logistic regression was used to calculate propensity scores, and Propensity Score Matching (PSM) was applied to control for inter-group confounding factors. The matching process adopted a 1:1 nearest-neighbor matching method with a sampling strategy of no replacement, and the caliper value was set at 0.1. Propensity scores were estimated based on potential variables such as relevant baseline characteristics and clinical features. After matching, the absolute standardized mean differences (SMD) of variables between the MACE group and the non-MACE group tended to be balanced. Using the criterion that the absolute value of SMD is less than 0.1, the covariates of the two groups were considered to achieve good balance after matching. Exclusion criteria were as follows: previous coronary revascularization, incomplete clinical data of patients, poor quality of CCTA images, abnormal coronary artery origins, coronary revascularization within six weeks of the CCTA examination, target plaque located in myocardial bridges, malignant tumors, and patients with other cardiac diseases (Figure 1). All enrolled patients were divided into training and test groups in a 7:3 ratio. The local institutional review board and ethics committee approved this retrospective study [approval number: Y2025004CS(KT)-004-01].

### 2.2 CCTA acquisition

CCTA scans were performed using a 256-slice CT scanner (Brilliance iCT, Royal Philips) with prospective electrocardiographic gating. The scanner specifications included collimator 128 × 0.625 mm, tube voltage 100 or 120 kV, automatic tube current adjustment, layer spacing 0.625 mm, layer thickness 0.9 mm, rotation time 270 ms. The acquisition time window was controlled at 30%–80% of the R-R interval. For optimal image quality, all patients received sublingual nitroglycerin to dilate the coronary arteries and a β-blocker (metoprolol, 25 or 50 mg, orally) was administered as needed 30 min before performing the CCTA scan to reduce the target heart rate of less than 65 beats per minute. A nonionic contrast medium (Iophorol 350 mgI/ml, Hengrui Pharmaceutical Co., Ltd., Jiangsu) was administered through a high-pressure syringe at a 5 ml/s flow rate through an anterior elbow vein, followed by 40 ml of saline at the same flow rate.

### 2.3 Segmentation and radiomics feature extraction for PCAT

PCAT automatic segmentation and radiomic feature extraction were performed using PCAT analysis tool software (Shukun Technology Co., Ltd.) (images were in a uniform DICOM format and de-noised as well as normalized before uploading them to the software). PCAT was defined as all adipose tissue surrounding the coronary arteries within a radial distance outside the vessel wall equal to the diameter of the vessel, with CT attenuation values ranging from −190 and −30 Hounsfield units (HU). To avoid the effect of aortic wall pulsation, we excluded PCAT in the RCA within 10 mm from the coronary ostium and automatically tracked PCAT in the proximal segment of the RCA (10–50 mm segment) by software.

**Figure 2:**
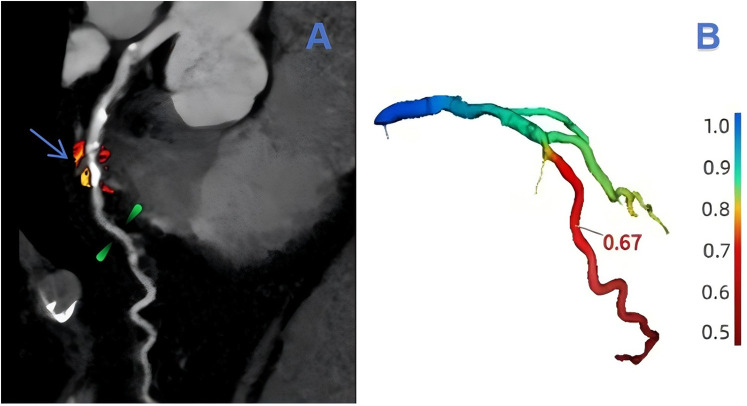
An example of target plaque selection in the left anterior descending branch (LAD, left anterior decending branch). In **(A)**, the blue-arrowed portion is the area of severe stenosis in the LAD, the green-marked portion is 2 cm from the area of severe stenosis, and the red and orange areas are the outlined extent of lesion-specific peri-coronary adipose tissue. The value of CT-FFR at 2 cm from the region of severe stenosis can be obtained in **(B)** as 0.67.

**Figure 3:**
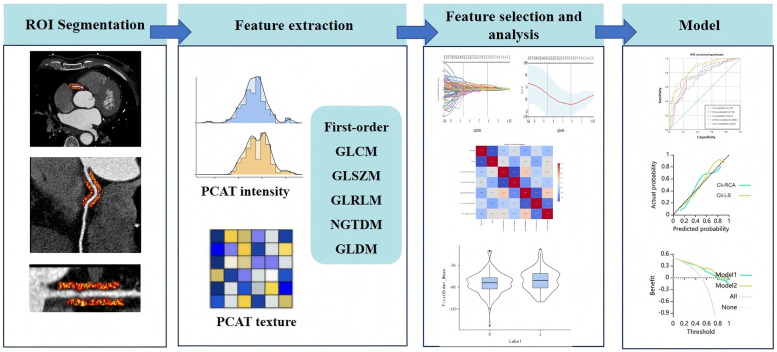
Radiomics workflow. PCAT, pericoronary adipose tissue.

The assessment of lesion-specific peri-coronary adipose tissue in patients with MACE was determined by target plaques, which were identified by CT-derived fractional flow reserve (CT-FFR) analysis (CoronaryDoc®-FFR, Shukun Technology, Beijing, China). A target plaque was defined as a lesion exhibiting a positive CT-FFR < 0.8, measured 2 cm distal to a plaque, indicating a potential impact on the hemodynamics of the corresponding coronary artery (as illustrated in Figure 2). When multiple target plaques existed, the plaque corresponding to the lowest CT-FFR value was selected as the target plaque. Because target plaques may not be present in non-MACE patients in the control group, we chose to perform PCAT segmentation and feature extraction at the narrowest point of the coronary artery in each non-MACE patient. Figure 3 shows the main processes of radiomics.

Each patient will undergo segmentation and extraction of PCAT radiomic features from the proximal RCA and around the target plaque. A total of 93 radiomic features will be extracted from lesion-specific PCAT or PCAT of the RCA for each segmentation (as detailed in the Supplementary Material), so that each patient will ultimately generate 186 radiomic features.

### 2.4 Feature selection and prediction model building

We plan to develop five risk prediction models to predict the occurrence of MACE in patients with coronary artery disease based on the training dataset: right coronary artery PCAT radiomics model (RCA-model), lesion-specific PCAT radiomics model (LS- model), clinical model (Cli-model), clinical-right-coronary-artery PCAT radiomics model (Cli-RCA model), and clinical-lesion-specific PCAT radiomics model (Cli-LS model). The performance of these models was evaluated using the test dataset. The process of feature selection and model construction is described below:

#### 2.4.1 Radiomics and clinical feature selection

##### 2.4.1.1 Clinical feature selection

Clinical features included were sex, age, BMI, total cholesterol, triglycerides, HDL cholesterol, LDL cholesterol, glucose, and C-reactive protein. Prior to feature selection, the clinical data were normalized, which was executed through Min-Max Normalization. This method involved a linear transformation of the data, scaling it to the range of [0,1], as calculated by the following equation:

$$X_{\mathrm{new}}\;=\;\frac{X-X_{\min}}{X_{\max}-X_{\min}}$$

Where *X* is the original data of clinical features, *X*_min_ is the minimum value in the feature dataset, and *X*_max_ is the maximum value in the feature dataset. Then, the Pearson correlation coefficient was used to screen for features with |*r*| > 0.95, so as to improve the efficiency and stability of the model. Finally, Lasso was used to screen clinical features with non-zero coefficients. Ten-fold cross-validation was used in the Lasso process to ensure good generalization performance of the Lasso regression, and clinical scores were calculated.

##### 2.4.1.2 Radiomics feature selection

The selection of radiomics features was consistent with the clinical feature screening process described in Section 2.4.1.1, and the radiomics score was calculated.

#### 2.4.2 Prediction model construction

It has been reported (15) that among multiple machine learning algorithmic models, the Extreme Gradient Boosting (XGBoost) algorithm performed the best in several studies and even outperformed or equaled the models specifically trained in each section. Therefore, this algorithm was adopted for all five models in this study. The optimal parameters of each model in the training set were determined through grid search and cross-validation, and the data in the test set were not involved in parameter tuning.

##### 2.4.2.1 Clinical model construction

The clinical model is an XGBoost model constructed based on clinical risk factors with coefficients other than zero selected by Lasso.

##### 2.4.2.2 RCA-model and LS-model construction

Both the RCA PCAT radiomic model and the lesion-specific PCAT radiomic model incorporated their respective radiomic features selected by Lasso regression analysis, and the models were constructed by XGBoost.

##### 2.4.2.3 Cli-RCA model and Cli-LS model construction

Both the Cli-RCA model and the Cli-LS model incorporated their respective Lasso-screened clinical risk factors as well as radiomic features selected after Pearson-Lasso regression analysis, and the models were constructed using XGBoost.

### 2.5 Model performance evaluation

The performance of the models was assessed using several indicator, including Area under the curve (AUC), accuracy, sensitivity, specificity, precision, and F1-Score. The DeLong test was used to compare the differences in AUC between different models. Calibration curves were plotted and the Hosmer-Lemeshow test was performed to assess the differentiation and calibration of the models. Clinical DCA was performed to assess the clinical utility of the models.

### 2.6 Statistical analysis

SPSS (version 26.0) software and Neusoft Explore Multimodal Medical Artificial Intelligence Platform were used for data analysis. Statistical tests were performed using two-sided tests, and *P* < 0.05 was considered a statistically significant difference. Categorical data were described by number and percentage, while continuous data that conformed to normal distribution were described by mean ± standard deviation, and continuous data that did not conform to normal distribution were described by median (interquartile spacing). Comparisons between groups of Categorical data were tested by χ^2^ test or Fisher's exact probability method, while the Students' t-test was used for normally distributed continuous data, and the Mann–Whitney U test was used for non-normally distributed continuous data.

## 3 Results

### 3.1 Clinical characteristics

Through PSM analysis, a total of 242 patients were successfully matched between the MACE group (*n* = 141) and the non-MACE group (*n* = 141) (Figure 1). Clinical characteristics of the training group (*n* = 164) and the test group (*n* = 78) are shown in Table 1. In the training and test groups, MACE patients and non-MACE patients were well matched with respect to sex, age, body mass index(BMI), cardiovascular risk factors, and medications, with no significant differences between the two groups (*P* > 0.05). The average duration between the CCTA examination and the occurrence of MACE was 15.7 ± 7.96 months. Based on the training group, total cholesterol (TC) and low-density lipoprotein cholesterol (LDL-C) were significantly associated with MACE in univariate analyses. Among the patients with MACE, there were 2 cases of cardiac death (1.65%), 4 cases of malignant arrhythmia (3.3%), 19 cases of nonfatal acute myocardial infarction (15.7%), 5 cases of new-onset congestive heart failure (4.8%), and 36 cases of coronary artery revascularization (29.75%), and 55 cases of readmission for unstable angina (45.45%).

**Table 1:** Clinical features in the training and test datasets.

Variables	Training			Test		
	MACE (*n* = 83)	Non-MACE (*n* = 81)	*P*-value	MACE (*n* = 38)	Non-MACE (*n* = 40)	*P*-value
Male	50 (60.24)	49 (60.49)	0.974	20 (52.63)	21 (52.50)	0.991
Age, years	63 (59, 68)	65 (59, 70)	0.189	63.9 ± 7.39	63.8 ± 6.88	0.966
BMI,kg/m2	25.6 ± 1.65	25.4 ± 1.37	0.549	25.8 ± 1.39	25.5 ± 1.36	0.304
Cardiovascular risk factors						
Smoking	27 (32.53)	25 (30.86)	0.819	14 (36.84)	12 (30.00)	0.522
Drinking	29 (33.73)	27 (33.33)	0.828	15 (39.47)	17 (42.50)	0.786
Hypertensive	51 (61.45)	51 (62.96)	0.841	24 (63.16)	25 (62.50)	0.952
Hyperlipidemia	9 (10.84)	8 (9.88)	0.839	3 (7.89)	3 (7.50)	0.948
Diabetes	29 (34.94)	30 (37.04)	0.780	13 (34.21)	15 (37.50)	0.762
Medications						
Beta-blocker	6 (7.23)	5 (6.17)	0.787	3 (7.89)	4 (10.00)	0.745
ACE-I/ARB	21 (25.30)	22 (27.16)	0.787	10 (26.31)	11 (27.50)	0.906
CCB	18 (21.69)	16 (19.75)	0.760	8 (21.05)	7 (17.50)	0.691
Statin	17 (20.48)	19 (23.46)	0.645	4 (10.53)	4 (10.00)	0.939
Antiplatelet	19 (22.89)	22 (27.16)	0.528	5 (13.15)	4 (10.00)	0.663
Inflammatory markers, Lipids and Hypoglycemia						
CHOL, mmol/L	5.24 (4.78, 5.62)	4.77 (3.86, 5.22)	<0.001*	5.14 ± 0.95	4.63 ± 0.99	0.023*
TG, mmol/L	1.85 (1.42, 2.7)	1.72 (1.38, 2.19)	0.226	1.77 (1.41, 2.51)	1.92 (1.69, 2.17)	0.296
HDL-C, mmol/L	1.12 (0.95, 1.34)	1.17 (0.94, 1.54)	0.101	1.15 (0.98, 1.27)	1.19 (0.99, 1.49)	0.365
LDL-C, mmol/L	2.91 ± 0.828	2.47 ± 0.874	0.001*	2.89 (2.16, 3.46)	2.34 (2.05, 3.15)	0.335
Blood glucose, mmol/L	6.07 (5.25, 7.03)	6.15 (5.33, 7.82)	0.859	5.87 (5.06, 6.88)	5.95 (4.96, 7.20)	0.968
C-reactive protein	4.8 (3.34, 6.55)	4.5 (3.25, 5.87)	0.406	4.25 (2.04, 4.97)	4.61 (3.88, 7.17)	0.072

MACE, major adverse cardiovascular events; BMI, body mass index; ACE-I, angiotensin converting enzyme inhibitor; ARB, angiotensin receptor blocker; CCB, calcium channel blockers; CHOL, total cholesterol; TG, triglycerides; HDL-C, high-density lipoprotein cholesterol; LDL, low-density lipoprotein cholesterol.

*Indicated *p* < 0.05 with significance.

### 3.2 Feature selection and construction of the model

#### 3.2.1 Clinical factor selection and model construction

Clinical features were screened by Lasso and showed that total cholesterol, high-density lipoprotein cholesterol, and low-density lipoprotein cholesterol were independent risk factors for MACE. Cli-model was constructed based on the independent risk factors screened above. The clinical score was calculated using the following formula:

$$\begin{array}{c} \mathrm{Cli}-\mathrm{Score}=0.4466988849281064-0.08799032630039208\\ \times \mathrm{CHOL}+0.0010963240852162903\\ \times \mathrm{HDL}-C+0.34643333392075903\times \mathrm{LDL}-C \end{array}$$

#### 3.2.2 Radiomics feature selection and model construction

After feature selection by Min-Max Normalization, Pearson correlation coefficient, and Lasso regression, the remaining radiomics features for lesion-specific PCAT and RCA were 13 and 4 (Supplementary Material). LS-model and RCA-model were constructed based on the radiomics features selected above. Radiomics scores for both were calculated using the formula:

$$\begin{array}{r} \begin{array}{c} \mathrm{RCA}-\mathrm{Score}\;=\;0.15369785450499812-0.14163992422547764\\ \times \mathrm{GLCM}\_\mathrm{InverseVariance}-0.26511991704637805\\ \times \mathrm{GLSZM}\_\mathrm{LargeAreaHighGrayLevelEmphasis}-0.6789869017416388\\ \times \mathrm{GLDM}\_\mathrm{DependenceNonUniformity}+0.8698829309013162\\ \times \mathrm{GLDM}\_\mathrm{DependenceNonUniformityNormalized} \end{array} \end{array}$$

$$\begin{array}{c} \mathrm{LS}-\mathrm{Score}\;=\;0.1002937208619752+0.21818380941201035\\ \times \mathrm{FirstOrder}\_90\mathrm{Percentile}+0.15214465516983042\\ \times \mathrm{FirstOrder}\_\mathrm{Mean}+0.9579877107143626\\ \times \mathrm{FirstOrder}\_\mathrm{Minimum}+0.40730891742443626\\ \times \mathrm{FirstOrder}\_\mathrm{TotalEnergy}+0.5738007754053269\\ \times \mathrm{GLCM}\_\mathrm{Imc}2-0.2924607299040573\\ \times \mathrm{GLCM}\_\mathrm{InverseVariance}-0.11191226949584672\\ \times \mathrm{GLSZM}\_\mathrm{SmallAreaLowGrayLevelEmphasis}\\ +0.040345574601068655\times \mathrm{GLSZM}\_\mathrm{ZoneEntropy}\\ -0.21375193026293665\times \mathrm{GLSZM}\_\mathrm{ZoneVariance}\\ +0.268626376677788\times \mathrm{GLRLM}\_\mathrm{ShortRunLowGrayLevelEmphasis}\\ +0.4697874003277172\times \mathrm{NGTDM}\_\mathrm{Coarseness}\\ +0.08654636959841888\times \mathrm{NGTDM}\_\mathrm{Strength}\\ -0.47909757307998235\times \mathrm{GLDM}\_\mathrm{GrayLevelNonUniformity} \end{array}$$

#### 3.2.3 Combined model construction

The clinical features and radiomic features selected by the above process were integrated with each other to construct Cli-RCA mode and Cli-LS model.

### 3.3 Model evaluation

**Figure 4:**
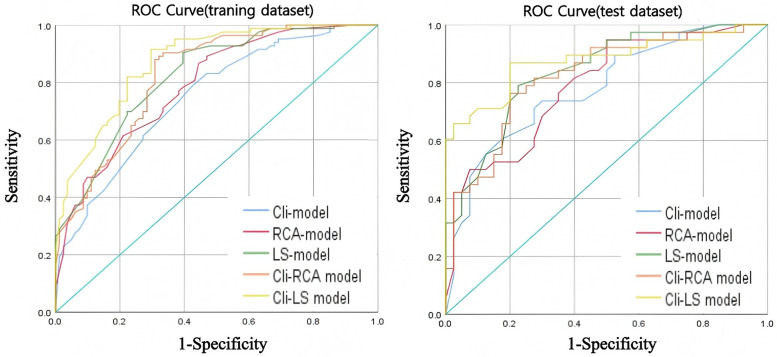
ROC curves for each model in the training dataset (left) and test dataset (right).

The ROC curves for the training and testing groups of each model are shown in Figure 4. Compared with the RCA-model [AUC = 0.789 (95% CI: 0.721–0.857), AUC = 0.788 (95% CI: 0.689–0.888)], the LS-model [AUC = 0.822 (95% CI: 0.759–0.884), AUC = 0.838 (95% CI: 0.751–0.925)] had superior predictive performance. The predictive performance of the clinical and radiomics features integration model was improved. Compared with the Cli-RCA model [AUC = 0.825 (95% CI: 0.762–0.887), AUC = 0.822 (95% CI: 0.729–0.915)], Cli-LS model [AUC = 0.873 (95% CI: 0.821–0.925), AUC = 0.877 (95% CI: 0.797–0.957)] still possessed a superior prediction performance for MACE. DeLong test showed that the training and test groups' AUC were not significantly different (*p* > 0.05). The AUC, accuracy, sensitivity, specificity, precision, and F1-Score of each model are detailed in Table 2.

**Table 2:** Performance evaluation of each model in the training dataset and test dataset.

Model	AUC (95% CI)		Accuracy		Sensitivity		Specificity		Precision		F1-score	
	Train	Test	Train	Test	Train	Test	Train	Test	Train	Test	Train	Test
Cli-model	0.748 (0.675, 0.822)	0.778 (0.678, 0.882)	0.652	0.654	0.891	0.737	0.407	0.575	0.607	0.622	0.722	0.675
RCA-model	0.789 (0.721, 0.857)	0.788 (0.689, 0.888)	0.654	0.695	0.771	0.605	0.617	0.725	0.674	0.677	0.719	0.639
LS-model	0.822 (0.759, 0.884)	0.838 (0.751, 0.925)	0.738	0.718	0.868	0.868	0.605	0.575	0.692	0.660	0.770	0.750
Cli-RCA model	0.825 (0.762, 0.887)	0.822 (0.729, 0.915)	0.744	0.756	0.771	0.763	0.716	0.750	0.736	0.744	0.753	0.753
Cli-LS model	0.873 (0.821, 0.925)	0.877 (0.797, 0.957)	0.781	0.833	0.819	0.868	0.741	0.800	0.764	0.805	0.791	0.835

95% CI, 95% confidence interval.

**Figure 5:**
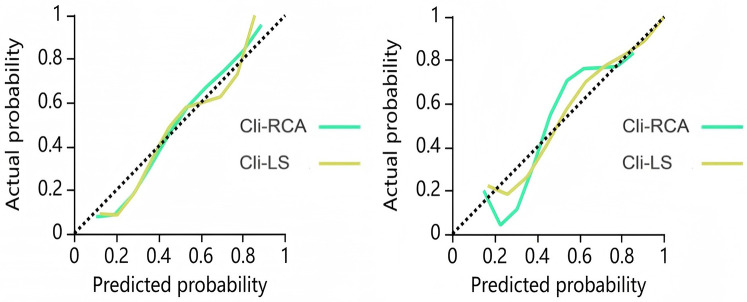
Calibration curves for the combined model training dataset (left) and test dataset (right). The closer the calibration curve is to the dashed diagonal line, the higher the calibration.

The calibration curves of Cli-RCA model and Cli-LS model are shown in Figure 5 (Hosmer-Lemeshow test, *P*-value > 0.05). This finding indicates that the above models have good agreement between the training dataset and the testing dataset, and the results of the prediction of MACE are reliable.

**Figure 6:**
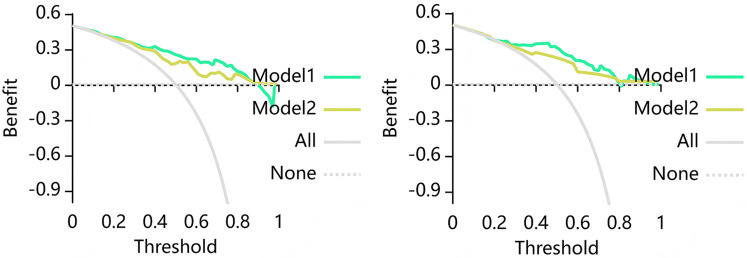
Decision curve analysis (DCA) for the combined model training dataset (left) and test dataset (right). Where Model 1 is the Cli-RCA model and Model 2 is the Cli-LS model. The DCA show that based on the training group, the Cli-LS model predicts higher benefits for MACE when the threshold probability is at greater than 30%, and based on the test group, the Cli-LS model predicts higher benefits for MACE when the threshold probability is greater than 22%. The *Y* axis denotes net benefit and *X* axis denotes threshold probability.

Clinical decision curve analyses were plotted to assess whether the model resulted in a higher net benefit to the patient. As illustrated in Figure 6, the DCA for the Cli-RCA model and the Cli-LS model indicates that the Cli-LS model possessed a higher overall net benefit within a reasonable threshold.

## 4 Discussion

A previous randomized controlled trial (16) demonstrated that in patients with stable coronary artery disease and functionally significant stenosis identified by FFR, FFR-induced percutaneous coronary intervention (PCI) plus optimal drug therapy reduces the need for emergency revascularization when compared to optimal drug therapy alone. This intervention is important for the prevention of end-point outcome events including the composite end point of death, myocardial infarction, or emergency revascularization. However, due to the invasive nature and high costs of FFR, its application in clinical practice has been limited. The exploration of a noninvasive and easy-to-use method for the prediction of MACE has become a focus of current research and a hot topic.

The relationship between coronary inflammation and high-risk plaque formation and rupture has been confirmed in past and recent studies (4). The rupture of plaque can lead to adverse clinical events. Common non-invasive circulating markers of inflammation include neutrophil-to-lymphocyte ratio and C-reactive protein-to-albumin ratio, and the elevation of these ratios not only predicts an increase in the severity of coronary heart disease, but also serves as an independent early warning signal of poor prognosis in patients experiencing ST-segment-elevation myocardial infarction, a finding that has been confirmed in several studies (17, 18). In addition, the triglyceride-glucose index has been identified as an independent risk factor for poor long-term prognosis in coronary heart disease (19). These biomarkers can be obtained by simple blood tests, and despite demonstrating non-invasiveness, convenience and high sensitivity in clinical applications. They are still deficient in specificity, which is similar to the Cli-model results of our study (refer to Table 1). ^18^Fluorine - Fluoride positron emission tomography, as a non-invasive imaging tool, is able to accurately capture the coronary artery inflammation, however, its application in actual clinical practice is severely constrained by its high cost and difficulty of access (20).

A correlation between CT attenuation and inflammation in PCAT has been reported (21) and the concept of CT fat attenuation index (FAI) was proposed. Oikonomou in the CRISP CT study (7) suggested that FAI measured around the RCA could be used as a representative biomarker for overall coronary artery inflammation. It was indicated that a high FAI value was also an important factor in increased cardiac mortality.

In another study (9), Oikonomou introduced an innovative imaging biomarker known as the fat radiomic profile (FRP), using new artificial intelligence techniques. The study revealed that, following a six-month treatment period for patients diagnosed with CAD, the FAI value around RCA elevated by inflammation was reduced, and the FAI value in the lesion area of patients with acute ST-segment elevation myocardial infarction was significantly reduced. The FRP was significantly elevated in patients with acute myocardial infarction compared with patients with stable coronary artery disease, but there was no significant change in FRP after 6 months of treatment. It suggests that FAI only detects dynamic reversible changes in PCAT components (e.g., aqueous phase, lipid phase interconversion), and this reversible change is based on the coronary inflammation produced. In contrast, the FRP also captured more advanced irreversible changes in PCAT (e.g., lipofibrosis and microvascular remodeling) in addition to inflammation, significantly improving the risk prediction of MACE. Collectively, these results suggest that radiomic features of PCAT can provide additional information beyond density resolution. Although the morphological features, radiomics features, and stability of high-risk coronary plaques are considered more direct predictors of MACE, the radiomics features of plaques require manual layer-by-layer delineation followed by semi-automatic segmentation and extraction, which is time-consuming and labor-intensive. Additionally, the reproducibility challenge of plaque radiomics has not been fully resolved (22, 23). In contrast, the PCAT in this study was automatically segmented and its radiomics features were extracted using a pericoronary adipose tissue analysis tool software (Shukun Technology Co., Ltd.). The delineation and extraction process is relatively simple and highly reproducible. Based on the aforementioned biological and methodological characteristics of PCAT, this study decided to adopt PCAT as the core biomarker.

Based on the series of studies by Oikonomou mentioned above, a study (13) confirmed the optimal predictive performance of the PCAT radiomics model of RCA for the prediction of MACE in the three major coronary arteries. PCAT radiomics characterization of lesion regions also outperformed traditional risk factors in MACE prediction (14). Prior research has established that lesion-specific FAI is more effective than FAI of RCA for predicting MACE risk (8). However, no prior studies have directly compared lesion-specific PCAT radiomics features with PCAT radiomics features of RCA for MACE prediction. Our research team undertook this comparison, and the findings indicated that the lesion-specific PCAT radiomics model was superior to the PCAT radiomics model of RCA in MACE prediction, and the predictive performance of the model was even better after clinical risk factors were added to the model, and the predictive performance of the Cli-LS model was still better than that of the Cli-RCA model. These results suggest that the lesion-specific PCAT radiomics feature may be a more representative risk predictor of MACE, which is consistent with the results of lesion-specific FAI in predicting MACE.

The reasons for the conclusions in the present study can be described as follows. Firstly, the PCAT of RCA is different from the ROI of lesion-specific PCAT, and the presence of non-diseased segments in the PCAT region of RCA 10–50 mm from the coronary ostium, and the radiomic features of the lesion-free region may affect the predictive performance of the model. Secondly, pericoronary lesion-specific PCAT may be a reliable indicator of local immune-inflammatory activation. A prior study (24) found an increased intracellular cytokine profile of pro-inflammatory cells in regions with high local FAI values around coronary arteries, which correlates closely with plaque susceptibility. In addition to inflammation, lipofibrosis and microvascular remodeling of PCAT in the lesion area may also be important factors for MACE development. Thridly, the clinical risk factors that we included in our model were total cholesterol, high-density lipoprotein cholesterol, and low-density lipoprotein cholesterol. The largest contributor to the downward trend in coronary heart disease deaths was the reduction in lipid levels (25). Therefore, elevated CHOL levels are pertinent for risk assessment and prediction of atherosclerotic disease. The inclusion of CHOL in the predictive model may improve the risk prediction performance of MACE, which is consistent with our findings. In addition, 13 radiomics features were selected in the lesion-specific PCAT radiomic model of this study, including four first-order gray-level statistics features, two gray-level Co-Occurrence Matrix (GLCM), three Gray-Level Size-Zone Matrix (GLSZM), one Gray-Level Run-Length Matrix (GLRLM), two Neighbouring Gray Tone Difference Matrix (NGTDM), one Gray Level Dependence Matrix (GLDM). Among them, the first-order gray-level statistical features provide fundamental statistics that quantitatively characterize the distribution of ROI intensity values, based on physical or functional measurements. GLCM, GLSZM, GLRLM, NGTDM and GLDM are heterogeneity-based and texture-based features to characterize spatial arrangement and local heterogeneity of the intensity values within an image (26, 27). The intensity-based first-order features and texture features may indirectly reflect the level of local inflammatory distribution in coronary arteries (e.g., the distribution and arrangement of adipocytes at the microscopic level). Whereas the heterogeneity features may reveal different components of adipose tissues or pathological changes (e.g., adipofibrosis, microvascular remodeling, and inter-conversion of the aqueous phase and the lipid phase of the adipocytes in PCAT). These features may reveal the development process of coronary atherosclerosis to a certain extent and provide more reference for the prediction of MACE.

Certainly, the possible association of high FAI with specific radiomic patterns in the vicinity of coronary lesions is not only a theoretical issue, but may also have important clinical implications. The study by Pan (28) developed a PCAT radiomic model to predict coronary plaque progression. The lesion-specific PCAT radiomic feature, with its more reliable and targeted nature, can predict offender plaque progression at specific locations, making prophylactic coronary stenting a future treatment option. The ORFAN study (29) demonstrated that in patients with non-obstructive coronary artery disease, FAI scores accurately capture clinical risk stratification as well as inflammatory risk beyond that explained by CCTA. In addition to coronary arteries, microvascular dysfunction may also be a background for cardiovascular MACE (30). That is, when CCTA shows a non-plaque outcome but with high-risk FAI or radiomic features, then specific anti-inflammatory therapy may be an option.

Our study has some limitations. Firstly, It was a single-center, retrospective study with a small number of cases and lack of an external validation cohort. Secondly, to control for confounding bias, we adopted 1:1 propensity score matching to construct the study cohort. This method resulted in a matched sample where the proportion of the MACE group was 50%, which is significantly higher than the actual prevalence of coronary heart disease in the real world. While this helps us estimate the association between predictors and outcomes more accurately, it may overestimate the discriminative ability (e.g., AUC value) of the developed models in the general consecutive enrollment population with a lower prevalence. Therefore, the performance of each model in this study should be regarded as the “potential performance” under its optimal conditions. Before applying these models to the real world, further external validation must be conducted in an independent cohort with representative prevalence to calibrate their predictive probabilities and re-evaluate their discriminative power. Thirdly, the CT scanning mode in this study was prospective adaptive scanning, and changes in tube voltage may have an impact on image quality, thus affecting radiomics feature extraction and selection. Fourthly, because the PCAT analysis software of Shukun Technology Co., Ltd. is capable of fully automatic and semi-automatic segmentation and feature extraction of PCAT of the right coronary artery and lesion-specific PCAT, allowing for the extraction 93 radiographic features at a time. Consequently, we assert that the radiomic features derived from Shukun's software exhibit a high degree of stability, and the intraclass correlation coefficient (ICC) consistency analysis was not performed before feature selection. However, fewer radiomic features per patient may affect the accuracy of the experimental results. Our team intends to utilize open-source software (e.g., 3D Slicer) to manually draw the ROIs of PCAT and extract the radiomic features, and compare the obtained experimental results with the current experiment. Fifthly, because the target plaques of lesion regions are determined by CT-FFR, when patients have multiple coronary lesions, the target plaque regions with CT-FFR <0.8, i.e., lesion-specific PCAT regions, may not necessarily be the regions with the most severe coronary inflammation. Therefore, the current study can only suggest that the radiomic profile of lesion-specific PCAT may be more effective than that of the right coronary artery PCAT in predicting MACE over a three-year period, without indicating a predictive advantage of lesion-specific PCAT in coronary inflammation. Future studies by our team will aim to explore and validate the FAI and lesion-specific FAI of the right coronary artery by including them in the same cohort of patients. Sixthly, the Region of Interest (ROI) for patients with MACE was defined based on the target plaque (CT-FFR < 0.8). For the control group, however, since patients without MACE may not have a target plaque, the ROI was defined based on the anatomically most stenotic site. The asymmetric ROI introduces potential confounding bias, which may affect the reliability of the results. In subsequent studies, our team will continue to expand the sample size, include a sufficient number of non-MACE patients who meet the target plaque criteria, and conduct further analyses. Lastly, the discussion of the radiomic features in this study has only been analyzed at the basic theoretical and clinical consequence level, and the intrinsic connection between these features and the biological properties of PCAT will be explored in depth in subsequent studies. We plan to validate the structural and functional changes of PCAT revealed by the radiomic features through biological experiments and molecular biology techniques, and further investigate the relationship between these changes and the development of coronary artery disease.

## 5 Conclusion

In summary, the lesion-specific PCAT radiomics model demonstrates a greater efficacy in predicting MACE compared to the PCAT radiomics model based on RCA. Incorporation of lesion-specific PCAT radiomic features into clinical cardiovascular risk factors could provide incremental predictive value.

## Data Availability

The raw data supporting the conclusions of this article will be made available by the authors, without undue reservation.
