# Supplementary material for: Radiomics model based on coronary CT angiography for predicting major adverse cardiovascular events in patients with coronary artery disease: comparison of lesion-specific pericoronary adipose tissue model and pericoronary adipose tissue model
Source: Front Cardiovasc Med. 2025 Oct 14;12:1600942. doi: 10.3389/fcvm.2025.1600942 (PMC12558845; doi:10.3389/fcvm.2025.1600942)
Supplement: Supplementary file 1 [file Datasheet1.docx]

Supplementary Material

# Supplementary Data

**Table S1.** List of radiomic features

| **Classification** | **Features (n=93)** |
| --- | --- |
| First Order Statistics | Energy, total energy, entropy, minimum, 10th percentile, 90th percentile, maximum, mean, median, interquartile range, range, Mean Absolute Deviation (MAD) , rMAD, Root mean squared, skewness, kurtosis, variance, uniformity |
|  |  |
| Gray Level Co-occurrence Matrix (GLCM) | Autocorrelation, joint average, cluster prominence, cluster tendency, cluster shade, contrast1, correlation, difference average, difference entropy, difference variance, joint energy, joint entropy, informational measure of correlation 1, informational measure of correlation 2, inverse difference moment, inverse difference moment normalized, inverse difference, inverse difference normalized, MCC, inverse variance, maximum probability, sum average, sum entropy, sum of squares |
| Gray Level Size Zone Matrix (GLSZM) | Small area emphasis, large area emphasis, gray level non-uniformity, gray level non-uniformity normalized, size zone non-uniformity, size zone non-uniformity normalized, zone percentage, gray level variance, zone variance, zone entropy, low gray level zone emphasis, high gray level zone emphasis, small area low gray level emphasis, small area high gray level emphasis, large area low gray level emphasis, large area high gray level emphasis |
| Gray Level Run Length Matrix (GLRLM) | Short run emphasis, Long run emphasis, gray level non-uniformity, gray level non-uniformity normalized, run length non-uniformity, run length non-uniformity normalized, run percentage, gray level variance, run variance, run entropy, low gray level run emphasis, high gray level run emphasis, short run low gray level emphasis, short run high gray level emphasis, long run low gray level emphasis, long run high gray level run emphasis |
| Neigbouring Gray Tone Difference Matrix (NGTDM) | Coarseness, contrast2, busyness, complexity, strength |
| Gray Level Dependence Matrix (GLDM) | Small dependence emphasis, Large dependence emphasis, gray level non-uniformity, dependence non-uniformity, dependence non-uniformity normalized, gray level variance, dependence variance, dependence entropy, low gray level emphasis, high gray level emphasis, small dependence low gray level emphasis, small dependence high gray level emphasis, large dependence low gray level emphasis, large dependence high gray level emphasis |

# Supplementary Figures

After the LASSO analysis, 13 and 4 features remained for lesion-specific area and RCA respectively (Figure.S2).

**
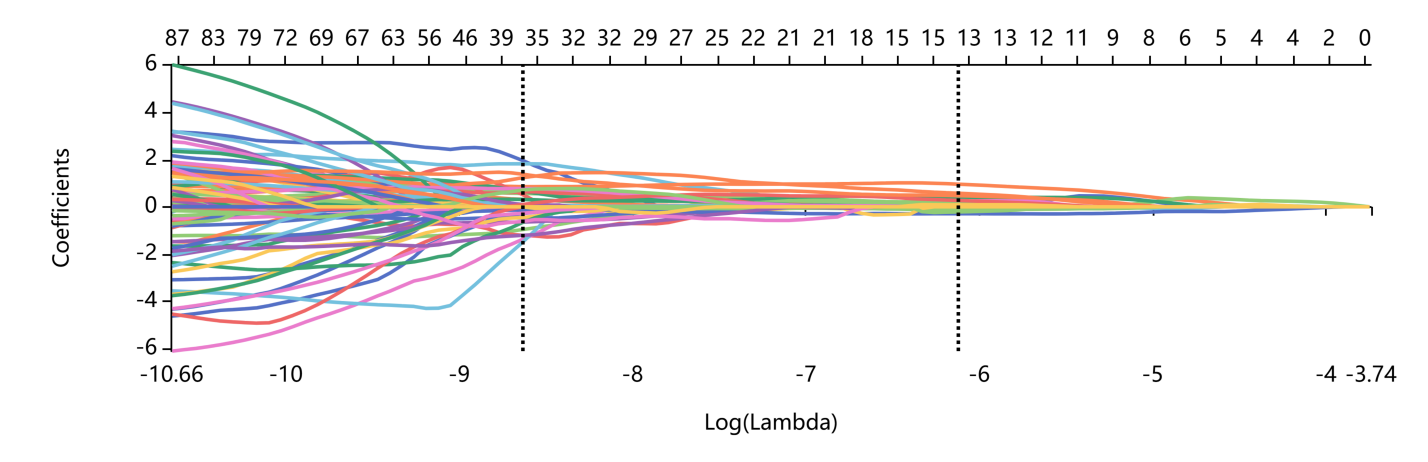
**

**Fig.S2a Path diagram of LASSO coefficients for lesion-specific area.**

**
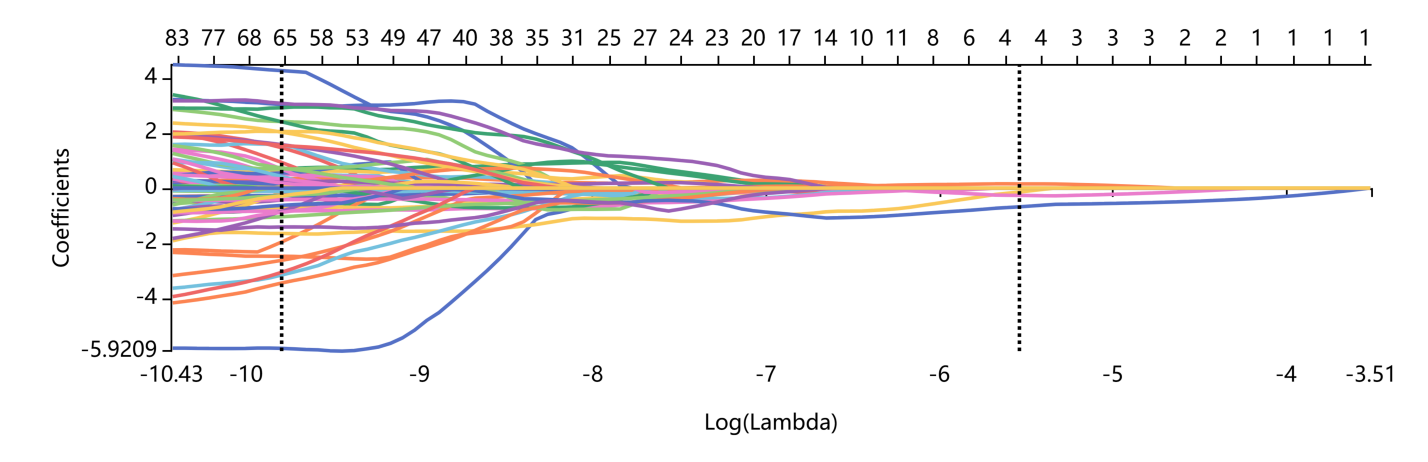
**

**Fig.S2b Path diagram of LASSO coefficients for RCA.**
